# The association of empathy and the work–family conflict in doctors of dental medicine

**DOI:** 10.2340/aos.v83.40852

**Published:** 2024-06-11

**Authors:** Lidia Gavic, Antonija Jerkovic, Vesna Ambarkova, Daniel Jerkovic, Antonija Tadin

**Affiliations:** a Study of Dental Medicine, School of Medicine, University of Split, Split, Croatia; b Department of Pediatric and Preventive Dentistry, Faculty of Dentistry, The Saints Cyril and Methodius University of Skopje, Skopje, North Macedonia; c Department of Maxillofacial and Oral Surgery, University Hospital of Split, Split, Croatia

**Keywords:** Empathy, work–family conflict, doctors of dental medicine

## Abstract

**Objectives:**

Work–family conflict is a growing problem worldwide, because of changing work–family demographic trends and the spread of technology. Empathy, as the ability to understand and share the feelings of other people, is the essential component of emotional intelligence that plays a crucial role in healthcare settings. This study aimed to assess the level of emotional empathy and investigate its relationship with work–family role conflicts among dental medicine doctors.

**Materials and methods:**

In this study participated 589 doctors of dental medicine from Croatia, Serbia, Bosnia and Herzegovina. The questionnaire included general and demographic data, the Emotional Empathy Scale questionnaire and the Work and Family Role Conflict Assessment Scale questionnaire.

**Results:**

The research results indicate no significant association between empathy and conflict between work and family roles (*R* = 0.032, *p* = 0.435). Women have achieved significantly higher scores in Emotional Empathy Scale (*p* ≤ 0.001), while there was no difference in Work and Family Role Conflict Assessment Scale according to gender (*p* = 0.194). A difference in emotional empathy was observed depending on where the respondents were employed (*p* = 0.045) and depending on the specialisation of the dental medicine (*p* = 0.021).

**Conclusion:**

Female doctors of dental medicine demonstrated higher emotional empathy while the work–family role conflict is experienced equally by both genders.

## Introduction

The modern lifestyle faced people with complex interpersonal relationships, overcrowded obligations and quick responses, which ultimately lead to tension and dissatisfaction [1].

The workplaces of the 21st century are dynamic and fast paced, bring a large number of advantages and opportunities but also carry excessive demands and pressure, affect an increase in stress levels, which can cause a decrease in success at work. The most common causes of stress in the workplace are the result of poor relations among staff and a lack of good communication. They are often the result of the inability to deal with one’s emotions, insufficient empathy and skills for working in a group [2]. Stress negatively affects several factors crucial for a successful organisation: concentration, creativity, innovation, movement coordination, decision-making process, communication and relationships, organisational climate, memory, job satisfaction, self-confidence and ultimately, the psychophysical health of employees [3]. Furthermore, stress at the workplace represents a global public health problem, is one of the biggest causes of occupational diseases and sick leave in the world and is among the leading economic problems of developed countries [4].

The conflict between work and family is a psychological phenomenon first determined by Greenhaus et al. in 1985 [5]. It is a conflict between roles that occur when the demands of energy, time or behaviour of the work role conflict with those of the family role and become incompatible [5, 6]. Work–family conflict is growing because of changing work–family demographic trends around the world, including increasing numbers of mothers with young children in the workforce; a sudden increase in the need for care for older people because of the ageing of the population and the increasing involvement of men in the demands of family care, particularly in developed Western countries. Also, it is also increasing because of the spread of technology and the prevalence of personal electronic communication devices that can keep individuals constantly connected to work and family concerns 24 h a day, 7 days a week [6].

Previous studies show a significant correlation between work–family life conflict and the level of emotional intelligence [7]. Employees who cannot balance work and family responsibilities will likely experience burnout. In the work environment, burnout is gradually becoming recognised as a severe challenge affecting individuals in human services, especially those in health services [8].

There are five elements of emotional intelligence: self-awareness, self-regulation, motivation, empathy and social skills [9]. Empathy, as the ability to understand and share the feelings of other people, is the essential component of emotional intelligence of social consciousness and is directly related to self-awareness [9, 10].

Relationships between doctors and patients are essential to every health profession, especially dental medicine. Patients often want a person-centred dentist, which makes the doctor more approachable [11]. A person-centred medical style requires the dentist to develop good communication and empathy [11]. Empathy plays a crucial role in healthcare settings. Namely, it can significantly encourage and motivate patients to cooperate during treatment. Ultimately, empathy leads to better outcomes and greater patient satisfaction.

This study aimed to determine the level of emotional empathy and to investigate the connection between emotional empathy and the incidence of conflict between work and family roles among doctors of dental medicine. Furthermore, this research examined the factors that can influence the degree of emotional empathy and the incidence of conflict between work and family roles among doctors of dental medicine. The hypothesis of this research was that empathy does not influence the incidence of conflicts between work and family roles among doctors of dental medicine.

## Materials and methods

This research involved doctors of dental medicine from the Republic of Croatia, the Republic of Serbia and Bosnia and Herzegovina. The research was in accordance with the Declaration of Helsinki and approved by the Faculty of Medicine Ethics Committee of the University of Split (Approval Class: 003-08/22-03/0003; Registration Number: 2181-198-03-04-22-0016) [12]. The questionnaire, an online survey created using *Google Forms*, was distributed to respondents via email and social networks. The completion of the questionnaire was entirely anonymous and voluntary. The questionnaire consisted of a total of 39 questions divided into three sections.

The first section of the questionnaire was related to the general and demographic data of the respondents. The second section of the questionnaire contained the Emotional Empathy Scale (EES). The EES measures the tendency to react emotionally to the emotional state of others. The scale contains 19 statements that describe emotional experiences that are consistent with the emotional state of others, as well as feelings of sympathy for those in trouble, with response option ranging from 0 to 4 (0 – does not apply to me at all, 1 – mostly does not apply to me, 2 – neither applies nor does it apply to me, 3 – mostly applies to me, 4 – completely applies to me). The highest possible score is 76 points; a higher score on the scale means a greater tendency to experience emotional empathy. The questionnaire was designed and validated in Croatian by Raboteg Saric [13]. The third segment of the questionnaire contained the Work and Family Role Conflict Assessment Scale (WFCAS). The WFCAS consisted of 12 questions with answers on a Likert scale from 1 to 7 (1 – Strongly disagree, 2 – Disagree, 3 – Somewhat disagree, 4 – Neither agree or disagree, 5 – Somewhat agree, 6 – Agree, 7 – Strongly agree). The statements are divided into two sets that differ in the direction of the disruptive influence (work to family and family to work). The first set of statements refers to the impact of business obligations on family life, while the second set refers to the impact of family obligations on the business environment. The total score is formed for each subscale separately by calculating the average value on the associated statements, and a higher score means a more significant conflict between work and family roles [14]. The questionnaire was translated and adapted into Croatian by Simunic et al. [15].

The research’s target population was dental medicine doctors from the Republic of Croatia, the Republic of Serbia and Bosnia and Herzegovina. According to the register of dental medicine at the Croatian Chamber of Dental Medicine, Serbian Dental Chamber and the Institute of Public Health of the Federation of Bosnia and Herzegovina, there are 5,066 doctors of dental medicine in Croatia, 9,412 in Serbia and 592 in Bosnia and Herzegovina [16–18]. Thus, the total number of doctors from the three mentioned countries was 15,070. With a 5% margin of error, a confidence interval of 90% and a response distribution of 50%, the minimum required sample size was 375.

### Statistical analysis

All correctly completed questionnaires were entered into the Microsoft Excel 2007 (Microsoft Corporation, Redmond, Washington, USA) program and, upon completion of the research, statistically processed with the help of the SPSS software package (IBM Corp., Armonk, New York). The descriptive statistics method was used to determine the basic statistical parameters (mean value, standard deviation, median and minimum and maximum values).

The distribution of responses within the EES and the WFCAS was evaluated using the Kolmogorov–Smirnov test.

To compare the statistical differences between the two groups, the T-test was employed while analysis of variance (ANOVA) was employed to compare the means among three or more groups. Additionally, Spearman’s correlation coefficient and linear regression analysis examined the relationship between respondents’ attitudes and socio-demographic characteristics. The significance level for all tests was set at *p* < 0.05.

## Results

Five hundred eighty-nine doctors of dental medicine participated in this research. The age range of the respondents in the study was from 24 to 71 years, with an average age of 35.71 ± 8.46.

Table 1 shows the results obtained by the respondents on the EES and the WFCAS. According to Bloom’s classification, on the scale of empathy, 288 (49%) respondents have high emotional empathy, 254 (43%) moderate and 47 (8%) respondents have weak emotional empathy. Furthermore, on the work and family role conflict scale, not a single respondent had the maximum number of points possible. According to Bloom’s classification, only one person has a high conflict between work and family roles, moderate 69 (12%) respondents and 519 (88%) respondents have a weak conflict between work and family roles. The demographic data of the respondents are presented in Table 2. Of the total number of respondents who work in private health practices, 144 (34.62%) stated that their practice has a contract with insurance companies. Of these, only eight respondents stated that it was a matter of some private insurance, while the rest had a contract with the state health insurance. No difference was observed, in the emotional empathy (*p* = 0.318) and conflict between work and family roles (*p* = 0.291) depending on the country doctors come from. According to the gender, the statistically significant difference was observed in empathy (*p* ≤ 0.001). Namely, women have achieved significantly higher scores in EES. Unlike that, there was no difference in conflict between work and family roles according to gender (*p* = 0.194) (Table 2).

**Table 1:** The results of the emotional empathy scale and the work and family role conflict assessment scale.

	Minimum	Maximum	Median (IQR)	Mean ± SD
EES	22	76	60 (13)	59.17 ± 9.38
WFCAS	12	69	37 (17)	36.58 ± 11.49

IQR: Interquartile range; SD: Standard deviation; EES: Emotional Empathy Scale; WFCAS: Work and Family Role Conflict Assessment Scale.

**Table 2:** The demographic data of the respondents and the score of emotional empathy scale and work and family role conflict assessment scale according to the demographics.

Variables	*N* (%)	EES	WFCAS
		Mean ± SD	Mean ± SD
Gender			
Male	110 (19)	53.33 ± 9.79	37.81 ± 11.82
Female	479 (81)	60.52 ± 8.76	36.30 ± 11.41
		*p* ≤ 0.001	*p* = 0.194
Country			
Republic of Croatia	255 (43.3)	60.14 ± 8.33	35.25 ± 11.36
Republic of Serbia	195 (33.3)	58.56 ± 9.54	37.84 ± 11.69
Bosnia and Herzegovina	139 (23.6)	58.28 ± 10.79	37.27 ± 11.28
		*p* = 0.318	*p* = 0.291
Dental specialty			
General dentist	467 (79.27)	59.34 ± 9.15	36.41 ± 11.32
Orthodontics	31 (5.26)	62.00 ± 9.50	37.39 ± 11.34
Periodontics	8 (1.36)	56.00 ± 6.21	36.75 ±12.22
Endodontics	16 (2.72)	59.69 ± 8.63	34.81 ± 8.32
Paediatric dentistry	24 (4.07)	60.25 ± 9.87	34.63 ± 11.42
Prosthodontics	20 (3.39)	58.35 ± 10.63	48.55 ± 9.33
Oral medicine	2 (0.34)	58.00 ± 5.66	27.55 ± 4.95
Oral surgery	21 (3.57)	51.76 ± 11.60	32.33 ± 13.52
		*p* = 0.021	*p* ≤ .001
Workplace			
Private practice owner	171 (29.03)	58.64 ±9.87	37.78 ± 11.95
Private practice part of the team	245 (41.60)	58.52 ± 9.5	37.17 ± 11.09
Health centres	126 (21.39)	59.8 ± 9.12	34.07 ± 11.51
Clinical Hospital centres	21 (3.57)	64.05 ± 6.67	36.29 ± 11.78
Polyclinics	26 (4.41)	61.44 ± 6.56	35.70 ± 10.72
		*p* = 0.041	*p* = 0.045

EES: Emotional Empathy Scale; WFCAS: Work and Family Role Conflict Assessment Scale.

A difference in emotional empathy was observed depending on where the respondents were employed (*p* = 0.045). A difference was observed between doctors of dental medicine who work in clinical hospital centres with those who work as part of a team (*p* = 0.006), in private practices as owners (*p* = 0.009) and doctors employed in health centres (*p* = 0.031). Furthermore, a statistically significant difference was observed in the conflict between work and family roles depending on where the respondents were employed (*p* = 0.041). The Tukey *post hoc* test showed a difference between employees who work in health centres and those who work in private practices as owners (*p* = 0.033) (Table 2).

Considering whether they are specialists in certain branches of dental medicine or general doctors of dental medicine, a statistically significant difference was observed according to the ANOVA in the empathy of the respondents (*p* = 0.021) (Table 2). The Tukey *post hoc* test showed a statistically significant difference between dental surgery specialists compared to non-specialist doctors (*p* = 0.007), orthodontic specialists (*p* = 0.003) and paediatric dental medicine (*p* = 0.048). In addition, a statistically significant difference was observed in the conflict between work and family roles concerning the specialisation in which the respondents are engaged (*p* ≤ 0.001). The difference was observed between specialists in dental prosthetics and specialists in orthodontics (*p* = 0.022), endodontics (*p* = 0.011), paediatric dental medicine (*p* = 0.002), oral surgery (*p* ≤ 0.001) and doctors who are not specialists (*p* ≤ 0.001).

The research results indicate no significant association between empathy and conflict between work and family roles (*R* = 0.032, *p* = 0.435).

## Discussion

The aim of this study was to assess the level of emotional empathy among dental medicine doctors. Additionally, the research investigated the association between emotional empathy and conflict between work and family roles. According to the research results, 49% of respondents show high emotional empathy, 43% moderate and only 8% show weak emotional empathy. Considering the respondents’ nationality, no statistically significant differences were found in the levels of emotional empathy. The study included a significant proportion of female respondents, with as many as 81% of the participants identifying as women. Indeed, there has been a noticeable global trend of increasing representation of women in dental medicine [19]. Namely, the research conducted in the United States provides specific data regarding the representation of women in dental medicine. According to the study, the percentage of women in dental medicine increased from 24.5% in 2010 to 29.8% in 2016. Furthermore, the research indicates that enrolment in dental medicine programs in the USA reached gender parity, with women comprising 50.5% of students in 2018 [20].

In this study, women showed a significantly higher emotional empathy score than men. These results are consistent with previous research showing higher emotional empathy in women providing dental care [21, 22]. According to a 2016 study by Hojat, several plausible explanations exist for gender differences in emotional empathy, such as social learning, genetic predisposition and evolutionary background [23]. The psychoanalytic and evolutionary theory of parental investment suggested that women, because of their biological role in childbirth and child-rearing, develop a higher degree of caring attitudes towards their offspring than men [24]. This theory recommends that the mother–child relationship forms the foundation for the potential differences in empathy levels between women and men. These differences in empathy may also extend to professional contexts, including healthcare, in relationships with colleagues or patients [25].

In this research, no significant correlation between empathy and the age of the participants was not found. Those results align with similar research conducted in the United States in 2019, which also did not observe any changes in the level of emotional empathy based on the age of doctors of dental medicine [24].

Empathy is crucial in fostering a strong dentist–patient relationship, and its importance is recognised across all dental specialties. However, some specialties may require a heightened level of empathic engagement, particularly in therapies that involve longer-lasting treatments, which naturally involve more frequent meetings and extensive consultations [11].

These lengthier therapies offer more opportunities for dentists to establish and nurture closer relationships with their patients. Dentists can better understand their patient’s needs, concerns and aspirations through regular interactions and comprehensive consultations [26].

Out of the total number of participants, 20.71% were either specialised in a specific dental branch or in the process of becoming accredited specialists in dental medicine. The study compared average empathy scores among doctors of dental medicine belonging to seven different speciality groups. The results revealed statistically significant differences in emotional empathy scores among doctors of dental medicine across different specialities (*p* = 0.014). *Post hoc* analysis showed a difference in emotional empathy scores between specialists in dental surgery compared to non-specialists (*p* = 0.007), specialists in orthodontics (*p* = 0.003) and specialists in paediatric dentistry (*p* = 0.048) (Table 2).

According to Gerlach, dental specialties are divided into ‘patient- or people-oriented’ specialities and ‘procedure- or technology-oriented’ [24]. ‘Patient- or people-oriented’ specialties typically focus on direct patient interaction, communication and providing comprehensive care that considers the individual’s overall well-being. These specialties may involve treatments such as paediatric dentistry, orthodontics or general dentistry where establishing a strong patient–dentist relationship, understanding patient needs and addressing emotional aspects of care are the most important. On the other hand, ‘procedure- or technology-oriented’ specialties tend to emphasise the technical aspects of dental procedures and may be more focused on specific interventions or treatments. Examples of such specialties may include oral surgery, prosthetics or endodontics where precise techniques and technological advancements are essential for successful outcomes [24]. Moreover, research conducted by Bailey in 2001 found that medical students planning to pursue careers in patient-centred specialities scored significantly higher on empathy than their peers who planned to pursue careers in procedure-centred specialities [25].

This research indicates that doctors of dental medicine employed in clinical hospital centres, health centres and polyclinics have a higher level of emotional empathy than those in the private sector (Table 2). Contrary to the results of our research, the results of a study conducted in South Australia on show almost identical levels of empathy in doctors working in clinics in the private sector and clinical hospital centres [27].

Our research aimed to investigate the relationship between empathy and work–family conflict and the impact of individual components on work–family conflict. Among the doctors of dental medicine included in our study, 88% exhibited a weak conflict between their work and family roles, while 12% experienced a moderate conflict. Interestingly, none of the respondents reported a high conflict between work and family roles. In previous studies, it was acknowledged that the perception, prevalence and consequences of work–family conflict vary across different cultures. The fact that this research did not identify any disparities in the level of conflict based on the nationality of dental medicine doctors (Table 2) can be easily explained by the fact that countries included in this research have very similar cultures and even were historically part of the same country for a long time.

Based on the theory of gender roles, according to the research of Efeoglu and Ozcan, a higher probability of conflict between work and family roles was found in women [28]. In a study conducted in Turkey, female doctors reported statistically significantly more conflict between work and family roles compared to male colleagues [29]. Also, in a study conducted in Hungary in 2008, female doctors reported a significantly higher average level and prevalence of conflict between work and family roles compared to men. Female doctors reported significantly less support from parents, spouses and peers compared to men [30]. Unlike the aforementioned studies, in this study no connection was found between work and family role conflict according to the gender (Table 2). The association between the conflict between work and family roles and the age of the respondents was not observed in this research (Table 2), which contrasts with the findings of the study mentioned above conducted in Turkey, where the conflict between work and family roles decreased with increasing age [28]. A similar outcome was reported in a study conducted in Germany, where it was suggested that younger doctors experience more stressors in their professional and family lives. However, as they grow older, these stressors diminish because of the development of coping mechanisms, reducing work–family role conflict. Young doctors are particularly sensitive to high-stress levels, given their relatively high responsibilities and limited control at work. The literature also indicates that dissatisfaction can arise from unfavourable working conditions in lower positions during the early stages of their careers. This period coincides with the phase when young doctors start families and face various demands related to their personal lives [31]. Additionally, a study conducted in China revealed that younger doctors devote more time and effort to their work, which can easily lead to increased conflicts in family life and work dissatisfaction [32].

Our research has established a significant difference in the conflict between work and family roles regarding specialisation. The highest level of conflict was observed between specialists of dental prosthetics (Table 2). In the study conducted in Saudi Arabia in 2022, it was discovered that the outcomes of aesthetic dental treatment have a significant psychological impact on patients [33]. Namely, patients who are unsatisfactory with the aesthetic procedures, leading to potentially harmful doctor–patient relationships and, consequently, occupational stress in doctors, resulting in symptoms such as fatigue, anxiety, irritability and depression, all contributing to medical errors [34]. Research conducted in China in 2012 shows that conflict between work and family roles is positively associated with emotional exhaustion among doctors. Namely, conflict is associated with burnout, which can decrease job satisfaction [34]. According to the study by Zeb et al., emotional intelligence shows a significant positive relationship with self-efficacy and a significant negative relationship with conflict between work and family roles [35]. Even this research did not find a direct association between the level of emotional empathy and conflict between work and family roles among the respondents, it is essential to highlight the significance of emotional intelligence, particularly empathy, in effectively managing such conflicts among medical professionals. In the future, initiatives can be undertaken to raise awareness about the importance of emotional intelligence in promoting the psychological well-being of healthcare workers. Also, each country should be developing national strategies to cope with workplace stress can also play a crucial role in preventing health issues of working population.

## Conclusion

Female doctors of dental medicine demonstrated heightened levels of emotional empathy compared to their male colleagues. Moreover, the research revealed that the challenge of balancing work and family roles is a common experience shared by individuals of both genders.

## Data Availability

The data that support the findings of this study are available from the corresponding author upon reasonable request.
